# Acute oral Chagas disease following armadillo blood ingestion: a case report

**DOI:** 10.1590/0037-8682-0078-2026

**Published:** 2026-09-25

**Authors:** Estefanía Gartner-López, Raúl Andrés Vallejo-Serna, Stephany Barbosa-Balaguera, Sebastián Ayala-Zapata

**Affiliations:** 1 Valle University, Cali, Valle del Cauca, Colombia.; 2 Sebastián de Belalcázar Clinic, Cardiology, Cali, Valle del Cauca, Colombia.

**Keywords:** Atrial fibrillation, Chagas disease, Foodborne diseases

## Abstract

A 70-year-old man from rural Colombia presented with new-onset atrial fibrillation, dyspnea, and palpitations. A peripheral blood smear revealed a *Trypanosoma cruzi* trypomastigote, which was confirmed by molecular testing. He reported ingesting armadillo blood 3 weeks earlier, identifying oral transmission as the likely route. Treatment with nifurtimox, oral metoprolol, and enoxaparin resulted in conversion to sinus rhythm and symptomatic improvement, although a parasitological cure could not be confirmed. Because the patient had several competing risk factors for atrial fibrillation-advanced age, long-standing hypertension, heavy smoking, and active small-cell lung carcinoma-a causal relationship with the infection could not be established. Acute Chagas disease was therefore proposed only as a possible, potentially reversible contributing factor. To our knowledge, this is among the few reported cases of acute oral Chagas disease presenting as reversible atrial fibrillation, highlighting an underrecognized cultural risk practice in non-endemic settings.

## INTRODUCTION

Chagas disease is an anthropozoonosis caused by *Trypanosoma cruzi*, the most relevant manifestations of which include cardiomyopathy and gastrointestinal involvement^1^. The infection comprises an acute phase lasting 8 to 12 weeks, followed by a lifelong chronic phase in the absence of effective treatment. Acute infection is rarely diagnosed because fewer than 1% of cases are symptomatic; nonetheless, acute cardiac involvement occurs in up to 50% of cases, with atrial fibrillation reported in 3.3%^2^. Beyond classical vector-borne transmission, oral transmission through contaminated food or beverages has been increasingly recognized^3^ and is associated with greater acute morbidity and mortality than vector transmission^4^. In Colombia, oral outbreaks accounted for 40.3% of acute cases between 2012 and 2019^5^.

A structured literature search was conducted of the Cochrane Library, LILACS, SciELO, MEDLINE, PubMed, and PubMed Central databases using the terms “Chagas disease,” “atrial fibrillation,” “oral transmission,” and “armadillo.” Only one previously reported case of atrial fibrillation in acute orally acquired Chagas disease was identified^2^, with transmission attributed to contaminated açaí. The present study appears to be the first report of transmission attributed to ingestion of armadillo blood, a culturally rooted practice in some rural Colombian communities.

We describe a case of acute orally transmitted Chagas disease associated with consumption of armadillo blood, presenting as reversible atrial fibrillation in an older patient with multiple comorbidities; we do so without claiming a definitive causal link between the infection and arrhythmia.

## CASE REPORT

A 70-year-old mestizo man from a rural area of the Colombian Pacific Region was admitted with progressively worsening dyspnea, including dyspnea at rest, palpitations, occasional chest discomfort, vomiting, and lower-limb edema during the week prior to admission. The patient had a history of arterial hypertension, smoking (56 pack-years), and small-cell lung carcinoma involving the entire left main bronchus (diagnosed 3 months earlier and not yet treated). He denied previously having cardiovascular symptoms. On admission, his heart rate was 140 beats per minute, and his blood pressure was 150/78 mmHg. Cardiac auscultation revealed irregularly irregular heart sounds; pulmonary auscultation revealed a diminished vesicular murmur in the left field; bimalleolar edema was present. The electrocardiogram captured on admission showed atrial fibrillation with a ventricular rate of 150 beats per minute (Figure 1).

**FIGURE 1:**
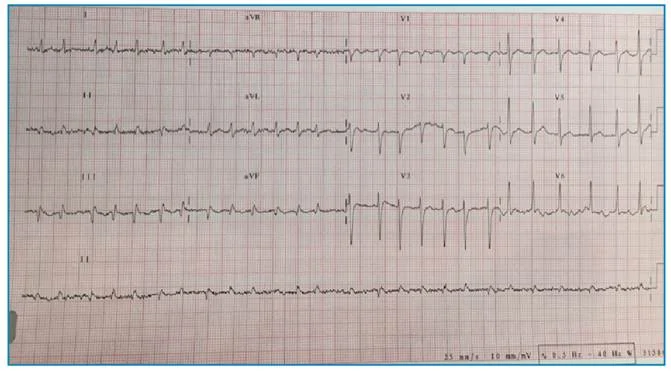
Admission 12-lead electrocardiogram showing atrial fibrillation with a ventricular rate of approximately 150 beats per minute.

The results of the laboratory tests conducted on admission showed mild normocytic, normochromic anemia, with normal leukocyte and platelet counts, normal serum electrolyte levels, preserved renal function, normal glucose levels, and normal thyroid function. The serum albumin concentration was low (2.1 g/dL), while total protein, corrected calcium, phosphorus, and magnesium levels were within reference limits. The troponin level was not measured because the patient declined further testing. The peripheral blood smear revealed a *T. cruzi* trypomastigote (Figure 2), and molecular testing confirmed the presence of *T. cruzi* DNA. A diagnosis of acute Chagas disease was established based on parasitological, molecular, and epidemiological grounds.

**FIGURE:**
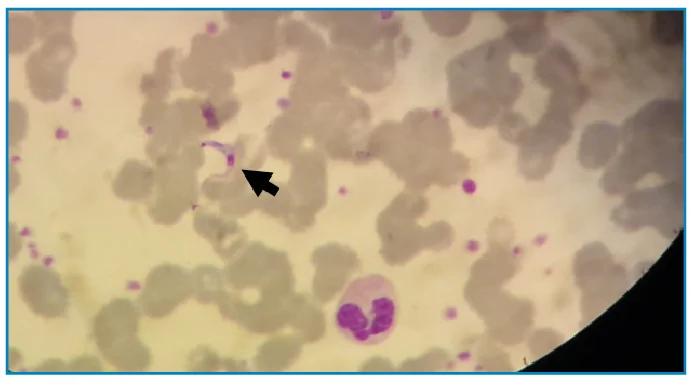
2: Peripheral blood smear (Wright-Giemsa stain) showing a trypomastigote of *Trypanosoma cruzi*.

Transthoracic echocardiography imaging showed normal cavity dimensions, a left ventricular ejection fraction of 52%, no valvular abnormalities, no signs of pulmonary hypertension or pericardial effusion, and a left pleural effusion. Contrast-enhanced chest computed tomography scans showed narrowing of the left main bronchus secondary to the previously known neoplasm, with complete atelectasis of the left lung and moderate to severe pleural effusion.

On directed questioning, the patient reported ingesting armadillo blood approximately 3 weeks before admission; such ingestion is a traditional practice in some rural Colombian communities, and believed to relieve respiratory symptoms^6^. This was identified as the most probable route of transmission.

Treatment was initiated with nifurtimox 10 mg/kg/day and oral metoprolol, while enoxaparin was administered for anticoagulation (CHA^2^DS^2^-VASc Score, 2). During hospitalization, conversion to sinus rhythm was observed on the electrocardiogram, with clinical improvement. After 1 week, the patient requested discharge to continue treatment of both the infection and the underlying neoplasm on an outpatient basis.

### Ethical Considerations

This case report was conducted in accordance with the ethical standards, and the study protocol was reviewed and approved by the institutional research ethics committee of the participating hospital (069-2025). Written informed consent was obtained from the patient for publication of the clinical details and accompanying images.

## DISCUSSION

Chagas disease is endemic in 21 Latin American countries and affects approximately 6 million people, with approximately 70 million at risk and 12,000 deaths annually^7^. Although vector-borne transmission predominates in rural endemic areas, oral transmission has become the leading mode in regions where vector-control measures have been effective, even in non-endemic areas^3^ ^,^ ^5^.

In Colombia, oral outbreaks have substantially increased the case-fatality rate of acute Chagas disease, reaching 7.9% in 2019-higher than that of other vector-borne conditions such as dengue and malaria^5^. Reported sources include açaí, sugarcane juice, palm wine and, less frequently, armadillo blood; our patient consumed this blood believing it would relieve dyspnea^6^.

Severe acute presentation occurs in fewer than 1% of infected patients and may include myocarditis, pericardial effusion, or meningoencephalitis^8^. Oral transmission is associated with higher acute morbidity and mortality than vector transmission^4^. In our patient, however, transthoracic echocardiography showed no features of acute chagasic myocarditis^9^: the cavity dimensions were normal; ejection fraction was preserved; and no ventricular dilation, wall-motion abnormality, atrial enlargement, or pericardial effusion were present. The absence of these findings argues against direct myocardial involvement by *T. cruzi* as the mechanism causing the arrhythmia; moreover, the troponin levels were not measured because the patient declined further testing, further limiting the assessment of myocardial injury.

Importantly, the patient had several well-established risk factors for atrial fibrillation that were independent of the infection. Advanced age is the single strongest risk factor for atrial fibrillation^10^, and our patient also had long-standing arterial hypertension and a history of heavy smoking (56 pack-years). Furthermore, he had active, untreated small-cell lung carcinoma, a malignancy associated with a markedly increased incidence of atrial fibrillation through systemic inflammation, a prothrombotic state, autonomic imbalance, and local mediastinal involvement^11^. The coexistence of these factors makes attributing the arrhythmia to acute Chagas disease alone impossible; at most, the infection can be regarded as a possible precipitating or contributing factor temporally associated with the episode.

Conversion to sinus rhythm coincided with antiparasitic and rate-controlling therapy and with resolution of the acute illness; this temporal sequence is consistent with a previous description of reversible atrial fibrillation in acute Chagas disease^2^, although spontaneous conversion of recent-onset atrial fibrillation is common and its presence does not, by itself, establish a causal link.

Compared with the only previous report of atrial fibrillation in acute oral Chagas disease^2^-which involved a young patient with açaí-related transmission-the present case differs in three respects: an older patient with significant comorbidity, transmission through ingestion of armadillo blood (a cultural rather than dietary practice), and confirmation of *T. cruzi* using both microscopy and molecular testing.

Recognizing the oral route as a current and increasingly important transmission mechanism, even in patients without exposure to vector-endemic areas, is essential for early diagnosis, appropriate antiparasitic treatment, and targeted preventive interventions, including education about culturally rooted high-risk practices.

This study had limitations inherent to a single case: the causal role of *T. cruzi* could not be confirmed, troponin levels and cardiac magnetic resonance imaging findings were not available, and the patient’s age and comorbidities provided plausible alternative explanations for the arrhythmia. These limitations should be considered when interpreting the proposed association.

Acute Chagas disease may be considered a possible, and potentially reversible, contributing factor to new-onset atrial fibrillation in patients from, or with epidemiological links to, endemic regions. However, in the present case, a definitive causal relationship could not be established because of the patient’s age and comorbidities. Identifying ingestion of armadillo blood as a culturally rooted practice in some Colombian communities is epidemiologically relevant and may guide early clinical suspicion and preventive interventions in similar settings.

## Data Availability

All relevant data supporting the findings of this case report are contained within the body of the manuscript and its figures.
